# Investigation of Innervation Zone Shift with Continuous Dynamic Muscle Contraction

**DOI:** 10.1155/2013/174342

**Published:** 2013-06-03

**Authors:** Ken Nishihara, Hisashi Kawai, Yu Chiba, Naohiko Kanemura, Toshiaki Gomi

**Affiliations:** ^1^Department of Physical Therapy, Saitama Prefectural University, 820 Sannomiya, Koshigaya, Saitama 343-8540, Japan; ^2^Tokyo Metropolitan Geriatric Hospital and Institute of Gerontology, 35-2 Sakaecho, Itabashi-ku, Tokyo 173-0015, Japan; ^3^Division of Sensory and Motor System Medicine, University of Tokyo, 7-3-1 Hongo, Bunkyo-ku, Tokyo 113-0033, Japan; ^4^Tokyo Ariake University of Medical and Health Sciences, 2-9-1 Ariake, Koto-ku, Tokyo 135-0063, Japan

## Abstract

Innervation zone (IZ) has been identified as the origin of action potential propagation in isometric contraction. However, IZ shifts with changes in muscle length during muscle activity. The IZ shift has been estimated using raw EMG signals. This study aimed to investigate the movement of IZ location during continuous dynamic muscle contraction, using a computer program. Subjects flexed their elbow joint as repetitive dynamic muscle contractions. EMG signals were recorded from the biceps brachii muscle using an eight-channel surface electrode array. Approximately 100 peaks from EMG signals were detected for each channel and summed to estimate the IZ location. For each subject, the estimated IZ locations were subtracted from the IZ location during isometric contractions with the elbow flexed at 90°. The results showed that the IZ moved significantly with elbow joint movement from 45° to 135°. However, IZ movement was biased with only a 3.9 mm IZ shift on average when the elbow angle was acute but a 16 mm IZ shift on average when it was obtuse. The movement of IZ location during continuous dynamic muscle contraction can be investigated using this signal processing procedure without subjective judgment.

## 1. Introduction

Surface electromyography (EMG) is a widely used tool to measure muscle contraction. The amplitude and frequency component of the EMG signal are investigated to evaluate muscle activation and fatigue in clinical use [1–3]. However, the EMG signal, recorded from surface bipolar electrodes around the innervation zone (IZ) location has a low amplitude and high mean frequency around IZ [4, 5]. It is, therefore, recommended that the electrodes should be located away from IZ [6, 7]. It is often assumed that isometric contractions in a fixed intensity stabilize EMG measurements under this condition because IZ location should not shift during the contractions [8]. Since IZ corresponds to the neuromuscular junction concentration, it can be identified using an electrode array as the origin of action potential propagation in isometric contraction [9–12].

However, IZ shifts with changes in joint angle [13]. For example, IZ of the biceps brachii is changed with different elbow joint angles during isometric contractions [14]. It is, therefore, necessary to determine the degree of IZ shift with changes in the joint angle to obtain proper EMG recordings during dynamic contraction. IZ shift has been estimated by investigator's judgment using raw EMG signals of isometric contractions at each angle [14, 15].

Dynamic contraction changes muscle length and causes movement of a body part. Muscles can shorten or lengthen during the dynamic contraction. It is difficult to identify the IZ location using raw EMG signals recorded by electrode array because changes in muscle length generate unsteady signals in continuous dynamic contraction signals for continuous changes in the recording condition and IZ location [16].

We have developed a method with computer program to investigate IZ by simplifying and clarifying the raw EMG signal during voluntary muscle contraction. This method enables instantly clear visualization of movements of IZ location during voluntary dynamic muscle contraction.

## 2. Materials and Methods

### 2.1. Subjects

Fourteen healthy adult men aged 21.2 ± 2.1 years (mean ± standard deviation) were enrolled in the study. The subjects were provided with detailed information of the experiment prior to enrollment. The study was approved by the Ethics Committee at Saitama Prefectural University.

### 2.2. Experimental Protocol

The subjects were secured in a sitting position with their right upper arm in the vertical position. The exercise was divided into two sessions, based on the static and dynamic contractions of muscle. For the static contraction session, the isometric maximal voluntary contraction (MVC) was measured for right elbow flexion with the joint angle at 90°. A dynamometer for isometric contraction (*μ* Tas F-1, Anima, Japan; operation range 0–980 N) was used to measure force. MVC was defined as the highest value of the three maximal contractions after preliminary training.

**Figure 1:**
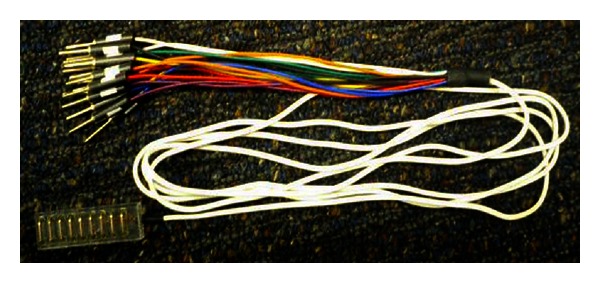
Surface electrode array used. Eight-channel electrode array, consisting of nine Ag/AgCl wires (width, 1 mm; length, 10 mm) attached at 5-mm intervals to a transparent acrylic resin box.

Surface EMG was recorded from the biceps brachii muscle. The eight-channel electrode array consisted of nine Ag/AgCl wires (width, 1 mm, length, 10 mm, Unique Medical, Tokyo, Japan) attached at 5-mm intervals to a transparent acrylic resin box (Figure 1). The two adjacent electrodes were used as a bipolar electrode. After cleaning the skin with alcohol, paste was used to attach the electrode array to the medial aspect of the belly of the right biceps brachii muscle along the direction of the muscle fibers. The electrode array was then secured to the skin with surgical tape, leaving sufficient distance between the electrode array and the proximal and distal tendons. The skin temperature was measured prior to the experiment using an infrared thermometer (TH03FH, Research Institute of Health Science and Education, Japan; error ±0.1°C) and was 33.2 ± 0.7°C.

During EMG recordings, all the subjects performed the contractions for 1 min with a 2 kg weight band load attached to the right wrist and the elbow flexed at 90° at a level below 10% of MVC for static contraction.

**Figure 2:**
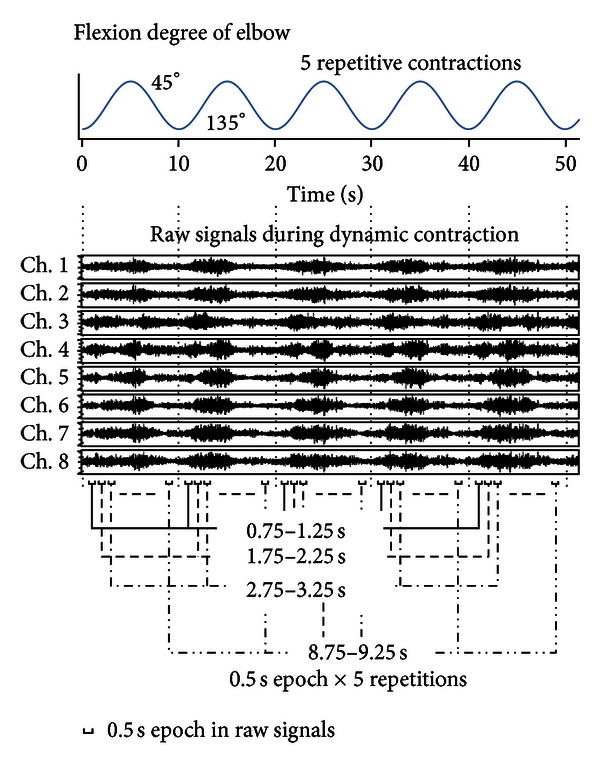
Example of EMG signals during dynamic contraction. The subjects' five repetitive contractions of their elbow joint angle between 45° and 135° with 5-s shortening and 5-s lengthening contraction cycles were recorded. The epochs analyzed were 2.5 s (each epoch of 0.5 s × 5 repetitions).

For the dynamic contraction session after more than three minutes of resting after the static contraction session, the subjects were asked to move their joint angle between 45° and 135° with 5 s shortening and 5 s lengthening contraction cycles with their forearms placed against the indexes to confirm the joint angle, with the same 2 kg weight band load attached to their wrist (Figure 2). The subjects performed the repetitive contractions seven to eight times. The timing of the movements was controlled by a blinking LED light synchronized with EMG recording.

The bioamplifier (MEG-6108, Nihon Kohden, Tokyo, Japan; CMRR, >80 dB; input impedance, 100 MΩ) had a 5–1000 Hz band-pass filtered prior to sampling. The data were sampled at a rate of 2,000 Hz, digitized with an analog-to-digital converter (NI 9215, National Instruments, Texas, USA), and stored on a computer.

### 2.3. Signal Processing

**Figure 3:**
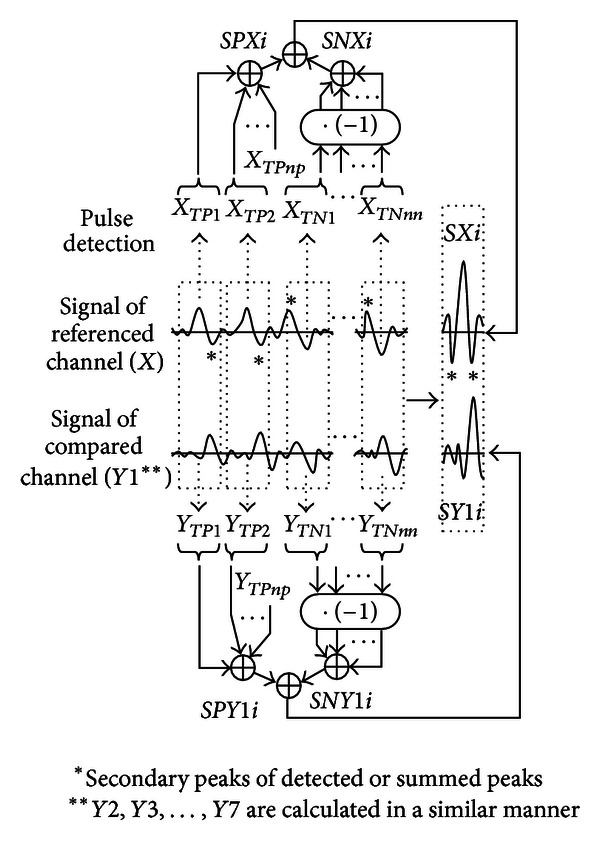
Schematic diagram of computation procedure. *TP*1, *TP*2,…, and *TP* *np*are the points of peaks detected by *X*. The time difference between *X* and *Y*1 is shown by the summed signals *SXi* and *SYi*. Notice that the secondary peaks before and after the summed peaks are smaller than the central peak in *SXi* (“∗” in *SXi*) because the summed signal of negative peaks are also summed after the reversed calculation (calculation of the signal “· (−1)”). The simultaneously summed signal from the compared channel (*SY*1*i*) shows a similar waveform, but the phase is shifted from *SXi*.

The signal processing of EMG data was composed of the static contraction session, to identify IZ location with elbow flexed at 90°, and the dynamic session, to identify the distance IZ moved from the IZ location during the static contraction. The EMG data were simplified before the estimation of IZ location. Pulses of positive peaks from a referenced channel were detected and summed (*SPXi* in Figure 3), and the same epochs of signals from compared channels were simultaneously summed (*SPY*1*i* in Figure 3). On the other hand, pulses of negative peaks from the referenced channel were also detected and reversed before summed (“· (−1)” of the detected pulses) and calculated the same as the pulses of positive peaks (*SNXi* and *SNY*1*i* in Figure 3). The summed signals of positive and negative peaks were summed together to generate a clear peak in each summed signal (*SXi* or *SYi*).

EMG data for the static contraction session were analyzed at 2.5 s periods centered at 25 s (23.75–26.25 s). We first summed the signal from the pulse detected for a single channel as the reference EMG (*X*). The pulse was detected using the waveform of channel 2, which was less influenced by irregular waveform near IZ or proximal tendon [17]. Thresholds were set to avoid detection of a noise component. When an increase in signal changed to a decrease and the increasing and decreasing amplitude and period were larger than the set thresholds in the EMG signal, the time point was registered as a peak. The peaks of the noise components from resting muscle EMGs were not detected by the threshold setting. In addition, the thresholds were adjusted for detecting around 100 pulses. The summed signal of positive peaks, including the increasing and decreasing slopes before and after the peaks at the time point *i* for reference *X* (*SPXi*), was calculated as follows (Figure 3):

(1) $$\begin{array}{c} SPXi=\sum_{i=1}^{np}X_{TPj+i}, \end{array}$$

where *i* (ms): −20, − 19.5, − 19,…, − 0.5, 0, 0.5,…, 19, 19.5, 20 (sample interval: 0.5 ms) over a 40 ms period. The peak of the summed signal was at 0 ms at the center of the time axis; *np* is the number of positive peaks detected from *X*, and *TPj* is the time of positive peak for pulse *j* detected from *X*. Namely, the detected peak times at n points were *TP*1, *TP*2, *TP*3,…, *TP* *np*.

The summed signal of negative peaks at the time point *i* in *X* (*SNXi*) was calculated using the following equation for reversal to a positive peak:

(2) $$\begin{array}{c} SNXi=\sum_{i=1}^{nn}\left(-1\right)\cdot \left(X_{TNj+i}\right), \end{array}$$

where *nn* is the number of negative peaks detected from *X* and *TNj* is the time of negative peak for pulse *j* detected from *X*. Namely, the detected peak times at *n* points were *TN*1, *TN*2, *TN*3,…, *TN* *np*.

The total summed signal of *X* (*SXi*) at the time point *i* was calculated as follows:

(3) $$\begin{array}{c} SXi=SPXi\;+SNXi. \end{array}$$

The summed signal of the accompanying channel (*Y*1) was then calculated as follows:

(4) $$\begin{array}{c} SPY1i=\sum_{i=1}^{np}{Y1}_{TPj+i},\\ SNY1i=\sum_{i=1}^{nn}\left(-1\right)\cdot \left({Y1}_{TNj+i}\right),\\ SY1i=SPY1i+SNY1i, \end{array}$$

where *np*, *nn*, *TPj*, and *TNj* were the same values as for *X*. Therefore, the time difference became more prominent compared to the reference EMG. *SY*2*i*, *SY*3*i*, *SY*4*i*,…, *SY*7*i* from the waveform of channels 1, 3, 4,…, 8 were calculated in a similar manner.

EMG data for the dynamic contraction session were analyzed for the 0.5 s epochs centered at 1, 2, 3,…, 9  (i.e., 0.75–1.25, 1.75–2.25,…, 8.75–9.25 s, resp.) after the beginning of every 5 repetitive shortening/lengthening contractions (Figure 2). The epochs analyzed were, therefore, 2.5 s (0.5 s × 5 repetitions). The joint angle intervals corresponding to each epoch time were changed for the natural smooth movement of the forearm based on the indexed angles. Approximately 100 pulses were detected from EMG data for the static and dynamic contractions for each subject.

### 2.4. Estimation of IZ Location

**Figure 4:**
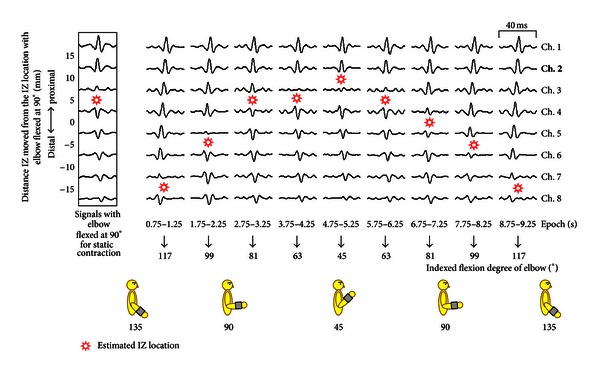
Example of the shift in the innervation zone (IZ) during dynamic contraction. EMG signal of channel 2 (bold) was selected as the referenced channel signal. The summed signals show the reversal in propagation direction by time shift of the peak of the signals. The summed signal over the IZ location shows the reversed peak in comparison with the referenced channel signal.

The former signal processing procedure was modified to estimate IZ [18]. The reversal in propagation direction from the peak of the calculated signal was plotted to identify IZ under the electrode array. EMG data for the static contraction were used to estimate the IZ location with the elbow flexed at 90°. EMG data for the dynamic contraction were used to estimate IZ locations by moving the joint angle. For each subject, IZ locations during dynamic contraction were subtracted from those during static contraction to show the distance IZ moved from the IZ location with static contraction (Figure 4). The IZ locations with this peak detection and summed methods were estimated at various elbow joint angles to investigate the reproducibility. We confirmed the differences among the estimated IZ locations by repeated trials and found the differences were smaller than 10%.

The software for the present study was developed using LabVIEW Ver. 2010 (National Instruments, Texas, USA).

### 2.5. Statistical Analyses

IZ movement was represented as a distance from the referenced location. The EMG data on the shifts in IZ location in different epochs was compared to the referenced location, and the IZ location between shortening and lengthening contractions, and also between static and each dynamic contraction, was compared using the paired nonparametric Wilcoxon matched-pair signed-rank test. *P* < 0.05 was considered statistically significant for all the comparisons. SPSS software version 17.0 (SPSS Japan, Tokyo, Japan) was used for all the statistical analyses.

## 3. Results

**Figure 5:**
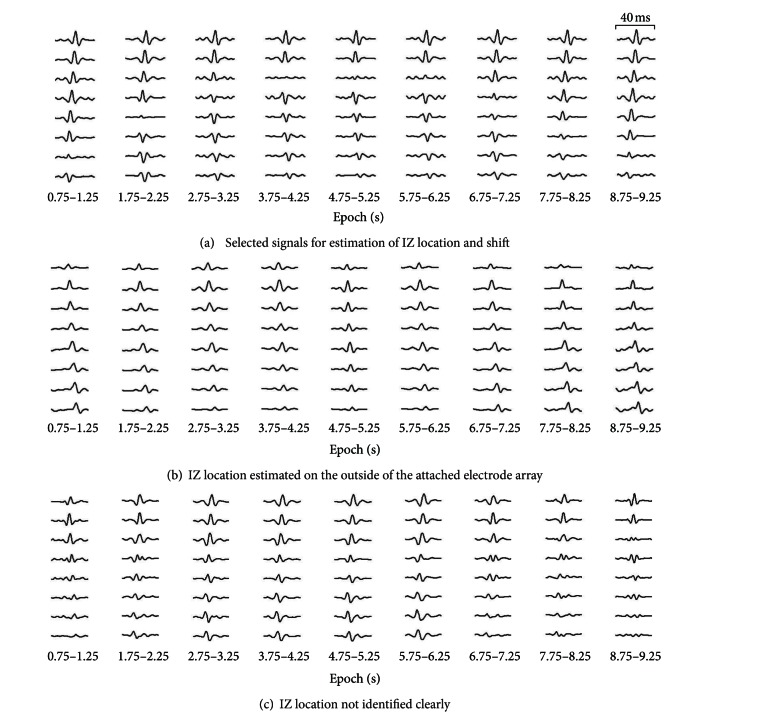
Examples of the selected signals for analysis (a) and not selected signals because the IZ location was estimated on the outside of the attached electrode array (b), and the IZ location was not identified clearly (c) during static contractions. Notice the peaks of the signals for each of (a), (b), and (c).

EMG data from 9 of the 14 subjects were selected for signal analysis. In the remaining five subjects, IZs were either located on the outside of the attached electrode array or not identified clearly during the static contractions (Figure 5).

A plot of an example of summed signals for a subject is shown in Figure 4. Reversal in propagation direction between signals was observed in estimated IZ locations during the elbow joint movement between 45° and 135°. IZ locations were assumed to be centered between neighboring signals. Extremely small amplitudes of summed signals were observed near IZ locations.

**Figure 6:**
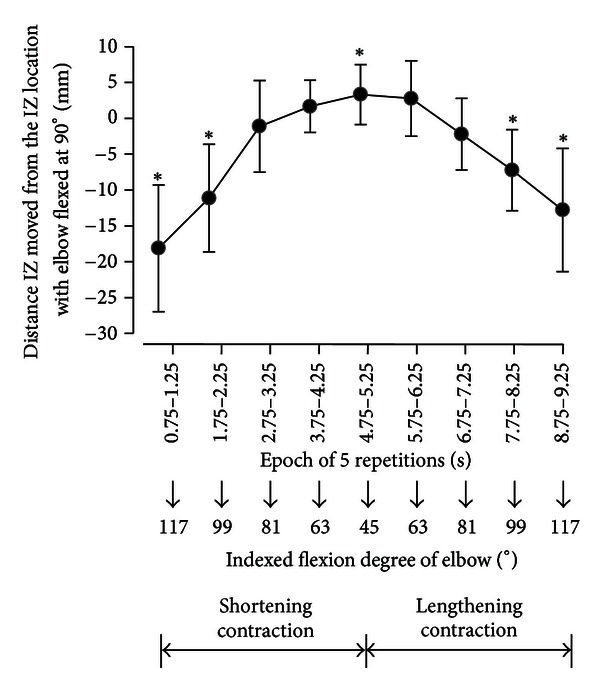
IZ locations shifted during dynamic contraction in the nine subjects. Data around an epoch of 0 were omitted because IZ locations were estimated on the distal side over the electrode array of channel 8 in the majority of subjects (six subjects). ∗ indicates the statistical significance of IZ location during dynamic contraction compared with that during static contraction with the elbow flexed at 90° (*P* < 0.05).

IZ locations in a moving elbow joint were averaged in the nine subjects (Figure 6). IZ locations demonstrated significant differences between different epochs of EMG data (ANOVA; *F* = 35.00, *P* < 0.01). The dynamic contractions can be classified as shortening and lengthening contractions. The epochs of 0.5 s length centered at 5 s (4.75–5.25 s) can be considered as the most acute joint angle around 45°. Paired epochs centered at 4 (3.75–4.25) versus 6 (5.75–6.25) s and 3 (2.75–3.25) versus 7 (6.75–7.25) s can be considered as having the same acute angles around 63° and 81°, respectively, and 2 (1.75–2.25) versus 8 (7.75–8.25) s and 1 (0.75–1.25) versus 9 (8.75–9.25) s can be considered as having the same obtuse angles around 99° and 117°, respectively, with shortening versus lengthening contractions (Figure 4). Significant differences were not observed between shortening versus lengthening contractions (Wilcoxon matched-pair signed-rank test).

Significant difference was observed between the estimated IZ locations during the static and dynamic contractions between epochs of 0.75–1.25, 1.75–2.25, 4.75–5.25, 7.75–8.25, and 8.75–9.25 s (Wilcoxon matched-pair signed-rank test; *P* < 0.05). 

IZ locations were estimated to move 20 ± 6.1 mm (mean ± standard deviation) with the elbow joint movement from 45° to 135°: 3.9 ± 3.3 mm with less than 90° and 16.1 ± 8.2 mm with larger than 90°.

## 4. Discussion

The major findings of this study are as follows: (1) IZ location from EMG signals during not only static contraction but also dynamic contraction can be estimated with this peak detection and summed methods; (2) the levels of IZ shift are changed according to the range of the joint angle.

### 4.1. Experimental Methods

All the subjects performed static contractions for one minute at a level below 10% of MVC. Because of this low-intensity exercise, minimal muscle fatigue would have influenced EMG signals during the static and dynamic contractions. However, it is important to note that the level of muscle contraction may have varied with the elbow joint angles in the dynamic contractions (amplitude of raw EMG in Figure 2). The thresholds had to be altered by the elbow joint movement.

### 4.2. Signal Processing Methods

In the present study, the thresholds of pulse detection were set to eliminate the noise component. However, the threshold value may have differed according to the subjectivity of the investigator, with more pulses being detected at smaller threshold values. Resting muscle EMG would need to be determined and thresholds set objectively. The summed signal was slightly altered with the number of summed pulses. However, the estimated IZ location did not change with the different summed pulses from 50 to 200 (data not shown). The stability waveform with an altered number of summed pulses was shown in a former report [11]. In the present study, the threshold would not have been influenced because of approximately 100 pulses being summed for the static and dynamic contractions of each subject.

Beck et al. compared the estimated IZ locations obtained from cross-correlation, the minimum amplitude of EMG, and maximum center frequency methods, and the superior accuracy of cross-correlation method was reported [19]. This peak detection and summed method would be easier for demonstrating IZ than cross-correlation. So, this method would be proper for investigating IZ location shift by dynamic contraction.

### 4.3. Estimated IZ Location during Dynamic Contraction

The results of this study indicated that the IZ location shifted significantly with movement of the elbow joint angle. The IZ location had a total shift of around 20 mm. However, it shifted 16 mm on average in the distal direction with the elbow joint shortening and lengthening contractions from 90° to 135° compared with a shift of only 3.9 mm on average in the proximal direction with the shortening and lengthening contractions 90° to 45° (Figure 6). Martin and MacIsaac investigated shifts in IZ for several joint angles of isometric contractions using loads equivalent to 20%–60% MVC [20]. A similar IZ shift was demonstrated for 20% MVC, the lowest level of muscle contraction investigated in their report as shorter than 5 mm shift with the elbow joint angles between 50° and 90° and 10 mm shift between 90° and 130°. This low-intensity contraction would be useful for elderly persons with lower physical strength. However, the smaller IZ shift with an acute angle was not observed for the higher level of muscle contractions. It is, therefore, necessary to investigate this bias in shift in greater detail in future studies.

The muscle was shortened and lengthened, which brought IZ shift relative to the electrodes used in this study. As shown in the raw signal in Figure 2, the level of muscle activities changed with the elbow joint angle [21].

Although the changes of EMG amplitudes by shortening and lengthening contractions were shown as the raw signal in Figure 2, significant differences of IZ shifts between shortening versus lengthening contractions were not observed in the contraction conditions used in this study. The 5-mm interval electrode array would be not enough to detect the IZ shift less than 5 mm in this study.

## 5. Conclusions

The current results suggest that EMG recordings from the biceps brachii muscle during low-level dynamic muscle contraction have to be performed under the consideration of the narrower IZ shift at acute angles from 45° to 90° rather than at obtuse angles from 90° to 135° in the elbow joint to avoid the influence of IZ in this experimental protocol.

The major advantage of these methods is that the movement of IZ location during continuous dynamic muscle contraction can be investigated clearly using this signal processing procedure.

The IZ shifts could not be estimated with the EMG data from 5 of the 14 subjects in this study. In the case of IZs located on the outside of the attached electrode array, the IZs would be identified for their IZ shifts by adjusting the attachment location or improving the electrode array by extending the length with more channels recorded.
